# Mismatch Negativity and P3a/Reorienting Complex in Subjects with Schizophrenia or At-Risk Mental State

**DOI:** 10.3389/fnbeh.2014.00172

**Published:** 2014-05-13

**Authors:** Yuko Higuchi, Tomonori Seo, Tomohiro Miyanishi, Yasuhiro Kawasaki, Michio Suzuki, Tomiki Sumiyoshi

**Affiliations:** ^1^Department of Neuropsychiatry, University of Toyama Graduate School of Medicine and Pharmaceutical Science, Toyama, Japan; ^2^Department of Neuropsychiatry, Kanazawa Medical University, Ishikawa, Japan; ^3^Department of Clinical Research Promotion, National Center Hospital, National Center of Neurology and Psychiatry, Tokyo, Japan

**Keywords:** mismatch negativity, reorienting negativity, event-related potentials, prodromal, schizophrenia

## Abstract

**Introduction:** We measured duration mismatch negativity (dMMN), P3a, and reorienting negativity (RON) in subjects with at-risk mental state (ARMS), patients with first-episode or chronic schizophrenia, and healthy volunteers. The main interest was to determine if these event-related potentials provide a biomarker associated with progression to overt schizophrenia in ARMS subjects.

**Methods:** Nineteen ARMS subjects meeting the criteria of the Comprehensive Assessment of ARMS, 38 patients with schizophrenia (19 first-episode and 19 chronic), and 19 healthy controls participated in the study. dMMN, P3a, and RON were measured with an auditory odd-ball paradigm at baseline.

**Results:** During the follow-up period (2.2 years), 4 out of the 19 ARMS subjects transitioned to schizophrenia (Converters) while 15 did not (non-Converters). dMMN amplitudes of Converters were significantly smaller than those of non-Converters at frontal and central electrodes before onset of illness. dMMN amplitudes of non-Converters did not differ from those of healthy controls, while Converters showed significantly smaller dMMN amplitudes compared to control subjects. RON amplitudes were also reduced at frontal and central electrodes in subjects with schizophrenia, but not ARMS. Converter subjects tended to show smaller RON amplitudes compared to non-Converters.

**Conclusions:** Our data confirm that diminished dMMN amplitudes provide a biomarker, which is present before and after the development of psychosis. In this respect, RON amplitudes may also be useful, as suggested for the first time based on longitudinal observations.

## Introduction

Schizophrenia is a disorder characterized by positive symptoms (hallucination, delusion, thought disturbance, etc.), negative symptoms (blunted affect, lack of volition, social withdrawal, etc.), and a range of disturbances of cognitive functions (Heinrichs and Zakzanis, 1998; Sumiyoshi et al., 2003; Harvey et al., 2004). In particular, cognitive impairment of schizophrenia is considered to largely determine the outcome of patients, including quality of life and social function (Green, 1996).

Prolonged duration of untreated psychosis (DUP) has been associated with poor long-term outcome, including work function, communication skills, and longer hospitalization (Loebel et al., 1992; Edwards et al., 1999; Malla et al., 2004; Melle et al., 2008; Yamazawa et al., 2008; Chang et al., 2011; Galderisi et al., 2012). On the other hand, shorter DUP has been related with a greater response to antipsychotic drugs in terms of symptoms and quality of life (Perkins et al., 2005). For these reasons, early detection, intervention, and treatment of schizophrenia are needed. In this context, it was reasonable that recent efforts have been directed to subjects with “at-risk mental state (ARMS)” or “ultra-high-risk patients” (McGorry et al., 2009).

The criteria for ARMS require that a young person aged between 14 and 30 years being referred for mental health difficulties met criteria for one or more of the following groups: (i) attenuated psychotic symptoms group (APS): have experienced sub-threshold, attenuated positive psychotic symptoms during the past year; (ii) brief limited intermittent psychotic symptoms group (BLIPS): have experienced episodes of frank psychotic symptoms that have not lasted longer than a week and have spontaneously abated; or (iii) trait and state risk factor group: have a first-degree relative with a psychotic disorder or the identified client has a schizotypal personality disorder, and they have experienced a significant decrease in functioning during the previous year (Yung et al., 1996; Broome et al., 2005).

To promote early diagnosis, objective markers, particularly those based on brain morphology, neurophysiology, and neuropsychology, have been reported to provide useful information (Nakamura et al., 2004; Kawasaki et al., 2007b; Higuchi et al., 2008, 2013b; Takahashi et al., 2011; Takayanagi et al., 2011; Lin et al., 2012). Accordingly, event-related potentials (ERPs) have been suggested to provide a biomarker for cognitive impairment of schizophrenia.

P300 (P3a and P3b) and mismatch negativity (MMN) have been widely used for this purpose. Specifically, patients with schizophrenia have been reported to show smaller P300 amplitudes compared with normal control subjects (Roth et al., 1980; Kawasaki et al., 1997; Bruder et al., 1998). Also, P300 amplitudes have been shown to be reduced in subjects with ARMS (Ozgurdal et al., 2008). On the other hand, P300 is affected by various factors, including medication (Umbricht et al., 1998; Higuchi et al., 2008, 2013a; Sumiyoshi et al., 2009), suggesting the utility as a state marker of psychotic disorders.

Mismatch negativity is another component of ERPs generated in response to occasional variations (e.g., duration, frequency, intensity) of acoustic stimuli, which occurs about 100–200 ms after the onset of deviant stimulation, with peak amplitudes at fronto-central leads (Naatanen et al., 2007, 2012). MMN amplitudes have been suggested to reflect pre-attentive cognitive operations, and decreased in patients with schizophrenia, as indicated by a recent meta-analysis reporting a large effect size (Umbricht and Krljes, 2005). Unlike the case with P300, MMN amplitudes are generally not affected by psychotropic drugs, for example, benzodiazepines (Kasai et al., 2002) and dopamine antagonists (Leung et al., 2007). For these reasons, MMN is considered to provide a trait marker for schizophrenia.

Duration mismatch negativity (dMMN) amplitudes have been shown to be reduced already in the prodromal stage of the illness (Bodatsch et al., 2011; Jahshan et al., 2012; Shaikh et al., 2012; Higuchi et al., 2013b). Furthermore, smaller dMMN amplitudes have been reported in subjects with ARMS who later converted to overt psychosis, compared to those who did not (Shaikh et al., 2012; Higuchi et al., 2013b). Thus, reduced dMMN amplitudes are regarded to predict conversion to schizophrenia in at-risk subjects (Sumiyoshi et al., 2013).

P3a is a positive waveform that appears following MMN, i.e., between 250 and 300 ms after the presentation of stimuli. Its amplitudes are largest at fronto-central electrodes. The P3a component is assumed to reflect a pre-attentive index of deviance detection, and represent the involuntary capture of attention (Friedman et al., 2001).

A negative activity reflecting attentional “re”-orienting follows P3a. This component is referred to as reorienting negativity (RON) (Schroger and Wolff, 1998), which peaks at latencies between 400 and 600 ms, and is centered on fronto-central electrodes (Schroger and Wolff, 1998; Otten et al., 2000; Schroger et al., 2000). The MMN/P3a/RON complex has been shown to provide a neurophysiological index of the cascade of three main processes involved in involuntary attention controls (i.e., automatic change detection, orienting of attention, and reorienting of attention), following deviant stimuli (Berti et al., 2004; Horvath et al., 2008).

Investigations into this series of ERP components should provide further insights into cognitive disturbances in schizophrenia spectrum disorders, which have not been satisfactorily addressed. Specifically, there is little information about the RON in schizophrenia spectrum disorders. Jahshan et al. (2012) measured the amplitudes of MMN, P3a, and RON complex, and found reductions of these parameters in schizophrenia patients. Also, amplitudes of MMN and P3a, but not RON were diminished in individuals at-risk for psychosis. In spite of the above cross-sectional study, further work is needed to test the utility of the ERP complex for predicting progression to schizophrenia in vulnerable individuals.

In this study, we measured dMMN, P3a, and RON amplitudes in subjects with ARMS, first-episode schizophrenia (FES), or chronic phase of the illness. These data were compared with those of normal control subjects. We also attempted to determine if these ERP parameters would predict later progression to schizophrenia in ARMS subjects by means of longitudinal observations. Specifically, preliminary data are provided on the evaluation of RON in relation to transition to overt schizophrenia in vulnerable subjects.

## Materials and Methods

### Participants

Diagnosis was made based on the Structured Clinical Interview for DSM-IV (SCID) for schizophrenia and the Comprehensive Assessment of At-Risk Mental State (CAARMS) for ARMS (Yung et al., 2005), by experienced psychiatrists. Most of these subjects were referred from Psychiatric Health and Welfare Center of Toyama (PHWCT), as previously described (Higuchi et al., 2013b). Nineteen ARMS subjects followed at the University of Toyama Hospital participated in this study [male/female = 9/10; mean (SD) age = 19.4 (3.6) years]. Thirty-eight schizophrenia patients also participated in this study. Patients with duration of illness <2 years were defined as FES [*n* = 19; male/female = 9/10; mean (SD) age = 22.8 (5.2) years], while those with duration of illness 2 years or longer were defined as chronic schizophrenia (CS) [*n* = 19; male/female = 9/10; mean (SD) age = 22.9 (3.6) years] (Higuchi et al., 2013b). The patients who allocated “first episode” are defined “single psychotic episode” and “duration of illness is <2 years.” CS patients are defined “duration of illness is more than 2 years.” Even if patients experienced only one psychotic episode, they allocated to CS group. We recruited normal control subjects from the community by advertisements. They are healthy volunteers [*n* = 19; male/female = 9/10; mean (SD) age = 19.4 (2.5) years] without any personal history of psychiatric illnesses, including schizophrenia or other psychotic disorders. All participants were right-handed. A psychiatric and treatment history was obtained from the subjects, families, and medical records. Subjects with a current history of substance abuse or dependence, seizure, or head injury were excluded from the study. Eligible patients had a complete physical examination and standard laboratory testing was normal. As clinical assessments, the Scale for the Assessment of Positive Symptoms (SAPS) and the Scale for the Assessment of Negative Symptoms (SANS) (Andreasen, 1990) were administered by an experienced psychiatrist. Demographic data at baseline evaluation are shown in Table 1A.

**Table 1:** **(A) Demographic and clinical data; (B) ERP data**.

(A)	Healthy controls (*n* = 19)	ARMS (*n* = 19)	First-episode schizophrenia (*n* = 19)	Chronic schizophrenia (*n* = 19)	Group comparison	
Male/female	9/10	9/10	9/10	9/10	n.s.	
Age (years)	19.4 (2.5)	19.4 (3.6)	22.8 (5.2)	22.9 (3.6)	*F*(3,74) = 4.94, *p* = 0.004	
Age of onset (years)	–	–	22.2 (5.2)	17.9 (3.9)	*p* = 0.007	
Duration of illness (years)	–	–	0.7 (0.6)	5.0 (2.3)	–	
Drug dose^a^	–	0.1 (0.4)	1.7 (2.0)	3.7 (4.2)	*F*(2,56) = 8.54, *p* = 0.001	
SAPS	–	17.3 (7.4)	27.0 (16.9)	19.2 (18.0)	*F*(2,56) = 2.29, *p* = 0.11	
SANS	–	60.8 (24.3)	60.6 (27.2)	53.3 (22.9)	*F*(2,56) = 0.52, *p* = 0.59	

**(B)**	**Healthy controls (*n* = 19)**	**ARMS (*n* = 19)**	**First-episode schizophrenia (*n* = 19)**	**Chronic schizophrenia (*n* = 19)**	**Analyze of variance (df = 3,75), group effect**	
					***F***	***p***

dMMN amplitude (μV)						
F3	−6.9 (1.7)	−6.2 (2.0)	−5.0 (1.8)	−4.6 (1.0)	7.505	<0.001**
F4	−7.5 (1.4)	−6.5 (2.2)	−5.2 (2.1)	−4.6 (2.0)	7.767	<0.001**
Fz	−7.4 (1.4)	−6.5 (2.0)	−5.4 (1.9)	−4.8 (1.5)	8.322	<0.001**
Cz	−6.0 (1.4)	−5.6 (2.1)	−4.8 (1.9)	−3.7 (0.9)	6.831	<0.001**
Pz	−4.2 (1.4)	−4.5 (4.0)	−3.3 (1.4)	−2.2 (0.9)	6.240	0.001**
dMMN latency (ms)						
F3	167.3 (15.1)	172.1 (17.5)	173.0 (23.0)	177.8 (30.7)	0.702	0.55
F4	169.1 (15.4)	175.8 (18.3)	172.5 (19.7)	176.6 (24.0)	0.404	0.75
Fz	172.2 (15.6)	177.3 (12.6)	173.0 (19.1)	176.8 (25.6)	0.364	0.77
Cz	168.4 (14.8)	182.3 (18.7)	174.6 (16.7)	177.6 (26.2)	1.675	0.18
Pz	173.0 (15.8)	188.6 (24.4)	178.3 (17.4)	174.8 (30.7)	1.753	0.16
P3a amplitude (μV)						
F3	1.6 (1.8)	1.1 (1.4)	1.3 (2.2)	1.5 (1.3)	0.277	0.84
F4	1.4 (2.3)	1.2 (1.8)	1.6 (2.1)	1.4 (1.4)	0.110	0.95
Fz	2.0 (2.3)	1.7 (1.5)	1.7 (2.2)	1.7 (1.1)	0.179	0.91
Cz	2.4 (2.4)	2.1 (1.6)	2.5 (2.1)	2.1 (1.4)	0.209	0.89
Pz	2.0 (2.2)	1.8 (1.4)	2.3 (1.8)	1.8 (1.3)	0.408	0.74
P3a latency (ms)						
F3	255.6 (23.5)	265.2 (25.0)	264.2 (28.3)	262.8 (23.0)	0.395	0.75
F4	256.9 (20.2)	269.7 (28.0)	262.9 (31.1)	255.2 (30.8)	0.755	0.52
Fz	254.6 (21.5)	268.7 (28.8)	261.8 (30.9)	262.3 (22.3)	0.314	0.81
Cz	255.1 (21.7)	266.0 (25.9)	254.8 (28.7)	262.2 (22.9)	0.509	0.67
Pz	255.7 (19.8)	272.2 (27.2)	254.7 (27.6)	261.4 (19.7)	0.359	0.78
RON amplitude (μV)						
F3	−4.4 (1.7)	−4.1 (1.7)	−3.5 (1.3)	−3.3 (1.3)	2.320	0.08
F4	−5.2 (1.8)	−4.2 (1.5)	−3.6 (1.7)	−3.4 (1.6)	4.191	0.009**
Fz	−5.1 (1.6)	−4.2 (1.8)	−3.9 (1.4)	−3.4 (1.7)	3.143	0.03*
Cz	−4.3 (1.9)	−3.8 (2.1)	−3.6 (1.6)	−3.2 (1.6)	1.143	0.33
Pz	−3.1 (1.8)	−2.7 (1.7)	−2.5 (1.5)	−2.6 (1.6)	0.391	0.76
RON latency (ms)						
F3	396.3 (51.8)	380.7 (49.4)	395.0 (42.7)	389.0 (52.6)	0.395	0.75
F4	392.7 (53.9)	404.0 (54.1)	409.1 (53.1)	385.4 (53.8)	0.755	0.52
Fz	396.2 (50.2)	397.2 (46.8)	409.3 (40.9)	397.7 (53.3)	0.314	0.81
Cz	401.2 (39.0)	398.3 (44.4)	412.5 (40.9)	397.0 (47.4)	0.509	0.67
Pz	409.5 (48.2)	401.7 (45.7)	411.7 (40.7)	397.4 (58.2)	0.359	0.78

*Values represent mean (SD)*.

*^a^ Risperidone equivalent (mg/day), ARMS, at-risk mental state; SAPS, Scale for the Assessment of Positive Symptoms; SANS, Scale for the Assessment of Negative Symptoms*.

*Values represent mean (SD). ARMS, at-risk mental state*.

*p < 0.05, **p < 0.01.

At-risk mental state subjects were followed-up at the hospital. Four out of the 19 ARMS subjects transitioned to schizophrenia during the observation period. When DSM-IV criteria were met, e.g., auditory hallucinations persisted or any delusion (for example, disturbance of the self) clearly observed, the subject was regarded to have converted to schizophrenia (Converters; Conv). Subjects who did not develop psychosis were defined as non-converters (Non-C). The average observation period for Non-C subjects was 2.2 ± 1.5 years.

### Electroencephalogram recording

Electroencephalograms (EEGs) were recorded based on the previous report from our laboratory (Sumiyoshi et al., 2006, 2009; Kawasaki et al., 2007a; Higuchi et al., 2008, 2010, 2013a,b; Itoh et al., 2011).

A 32-channel DC-amplifier (EEG-2100 version 2.22J, Nihon Kohden Corp., Tokyo, Japan) was used. Recordings were performed using an electro cap (Electrocap Inc., Eaton, OH) in a sound-attenuated room. Data were collected with a sampling rate of 500 Hz. EEG data were collected from 29 scalp electrodes (Fp1, Fp2, F3, F4, F7, F8, FC3, FC4, C3, C4, T3, T4, CP3, CP4, TP7, TP8, P3, P4, T5, T6, O1, O2, FPz, Fz, FCz, Cz, CPz, Pz, and Oz according to the extended International 10–20 system). All electrodes were referred to the average amplitude of the ear electrodes (bandwidth = 0.53–120 Hz, 60 Hz notch filter). Electrode impedance was <5 kΩ.

Measurements of dMMN/P3a/RON complex were based on our previous report (Higuchi et al., 2010). One-thousand auditory stimuli were delivered binaurally through headphones with inter-stimulus intervals 500 ms. Standard/target tones of 50/100 ms duration were randomly presented with the presentation probability of 0.9/0.1. All tones were 60 dB, 1000 Hz, and with a rise–fall time of 10 ms. The subjects were requested to watch silent animation movie (Tom and Jerry) and pay attention to the monitor and ignore the tones.

Averaging of ERP waves and related procedures were performed using Vital Tracer and EPLYZER II software (Kissei Comtec, Co. Ltd., Nagano, Japan). Epochs were 600 ms, including a 100 ms pre-stimulus baseline. Eye movement artifacts (blinks and eye movements) were manually rejected. MMN waveforms were obtained by subtract standard waveforms from target ones. MMN, P3a, and RON peaks were identified within the 150–250 ms (minus peak), 200–350 ms (plus peak), and 250–500 ms (minus peak) search windows, respectively.

### Statistical methods

Statistical analyses were performed using the Statistical Package for Social Sciences (SPSS) version 20 (SPSS Japan Inc., Tokyo, Japan). We performed comparison of age between four groups (HC, ARMS, FES, and CS) by one-way analysis of variance. Onset age and duration of illness of two schizophrenia groups (first-episode and chronic) were compared by independent *t*-test. Drug dose, SAPS, and SANS score among three groups (ARMS, FES, and CS) were analyzed by one-way ANOVA.

**Figure 1:**
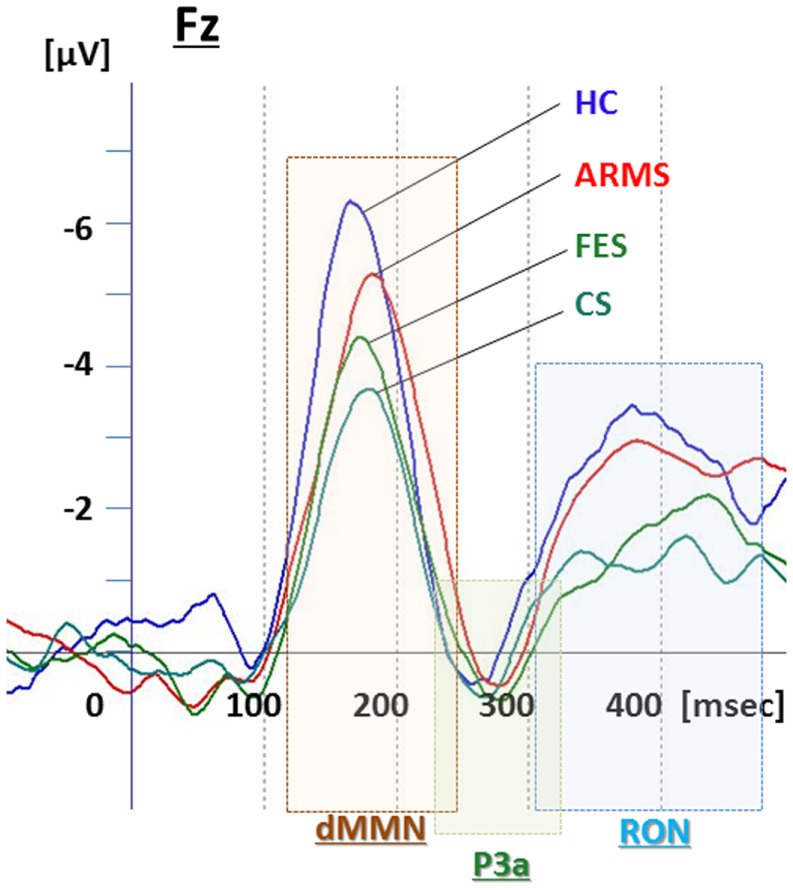
**Duration mismatch negativity (dMMN), P3a, and reorienting negativity (RON) complex waveforms at Fz for all subjects**. Waveforms are presented for healthy controls (HC, blue line), at-risk mental state (ARMS, red line), first-episode schizophrenia (FES, light green line), and chronic schizophrenia (CS, dark green line).

**Figure 2:**
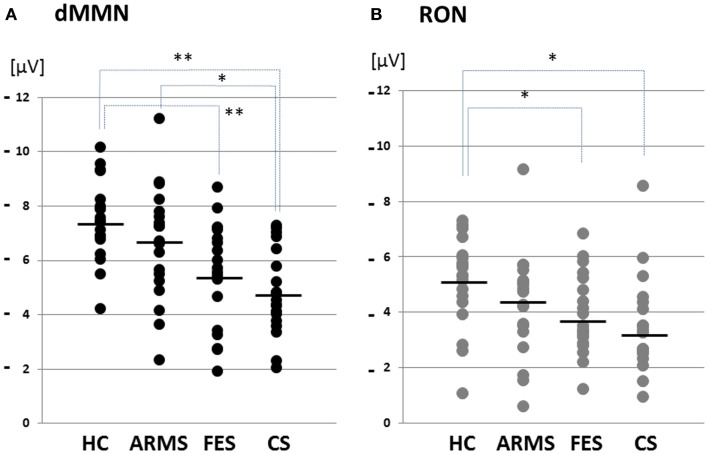
**Distribution of amplitudes of duration mismatch negativity [dMMN; (A)] and reorienting negativity [RON; (B)] at Fz for all subjects**. Data are presented for healthy controls (HC), ARMS, first-episode schizophrenia (FES), and chronic schizophrenia (CS). *p < 0.05, **p < 0.01.

**Figure 3:**
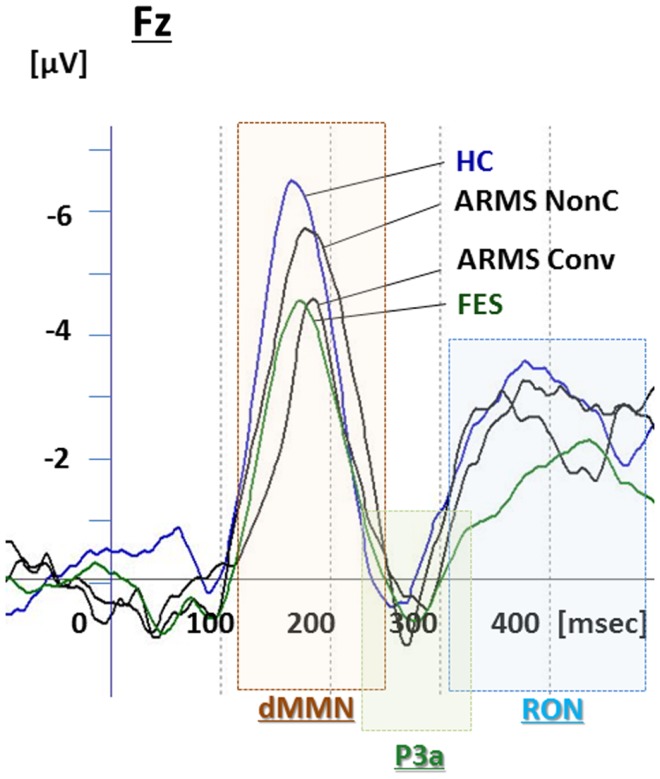
**Duration mismatch negativity (dMMN), P3a, and reorienting negativity (RON) complex waveforms at the Fz lead**. Waveforms are presented for healthy controls (blue line), ARMS Converters (Conv), and non-Converter (Non-C) (black lines), and FES (green line).

**Figure 4:**
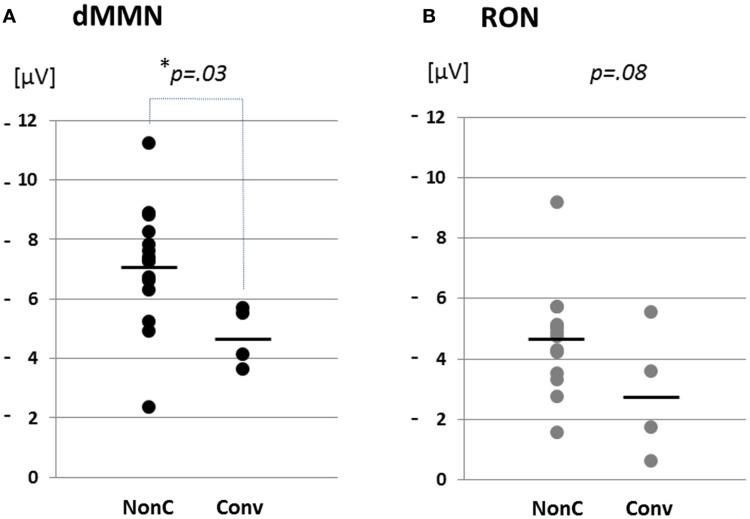
**Distribution of amplitudes of duration mismatch negativity [dMMN; (A)] and reorienting negativity [RON, (B)] at Fz for ARMS**. Data are presented Converters (Conv) and Non-Converter (Non-C). *p < 0.05.

Event-related potential amplitudes and latencies were measured and analyzed at five electrodes; three from frontal lobe (F3, F4, and Fz), and two from midline (Cz and Pz). They are typical electrodes that commonly used on ERP studies. MMN amplitudes are generally largest at frontal electrodes, so we choose three electrodes from frontal lobe. Moreover, grand average waveforms (Figures 1 and 3) and scatterplots (Figures 2 and 4) were drawn and analyzed by Fz lead as a representative of electrodes because amplitudes ERPs of Fz were largest. Laterality of ERPs was analyzed by F3/F4 comparison as we performed in previous report (Higuchi et al., 2008), but there were no difference in this study (data not shown).

Two-way ANOVA was conducted on amplitudes and latencies of dMMN, P3a, and RON, with “Stage” (HC, ARMS, FES, and CS) and “Lead” (F3, F4, Fz, Cz, and Pz) as fixed factors. Main effects (of Stage and Lead) were described on Table 1B (significant differences were seen in all leads of dMMN amplitude and F4/Fz of RON amplitude). The Stage-by-Lead interactions on amplitudes (dMMN, *F* = 1.172, *p* = 0.30; P3a, *F* = 0.511, *p* = 0.90; RON, *F* = 1.024, *p* = 0.42) and latencies (dMMN, *F* = 1.254, *p* = 0.246; P3a, *F* = 1.475, *p* = 0.13; RON, *F* = 0.516, *p* = 0.904) were not significant.

Gender difference between Conv and Non-C were analyzed by Chi-square test. Other factors (age, drug dose, SAPS, SANS, ERP amplitude, and latency) of them were calculated by independent *t*-test. All analyses of variance were corrected by Bonferroni correction.

Correlations of symptoms and ERP amplitudes were performed by Pearson product–moment correlation coefficient. SAPS scores (hallucinations, delusions, bizarre behavior, and positive formal thought disorder) and SANS scores (affective flattening/blunting, alogia, avolition-apathy, anhedonia-asociality, and attention) were used.

Raters were not informed of subjects’ profiles and diagnosis.

## Results

### Subjects’ profile

Demographic and clinical data of participants are shown in Tables 1A and 2. There was significant group difference in age [*F*(3,74) = 4.94, *p* = 0.004, ANOVA], and Conv subjects were older than Non-C in age (*p* = 0.009, *t*-test). Male/female ratio did not differ between of Conv. and Non-C groups [χ^2^ = 2.47, *p* = 0.3, Chi-square test].

**Table 2:** **Comparison between converters and non-converters of ARMS subjects**.

	ARMS (*n* = 19)		Group comparison (*p*)
	Non-C (*n* = 15)	Conv (*n* = 4)	
Male/female	7/8	3/1	χ2=2.47, *p* = 0.3
Age (years)	18.3 (2.2)	23.4 (4.9)	0.009
Drug dose^a^	0.1 (0.2)	0.4 (0.6)	0.12
SAPS	15.3 (7.0)	22.7 (5.8)	0.08
SANS	56.9 (26.3)	73.7 (9.6)	0.24

dMMN amplitude (μV)
F3	−6.5 (2.1)	−4.9 (0.6)	0.16
F4	−7.0 (2.2)	−4.6 (0.9)	0.06
Fz	−7.0 (2.0)	−4.7 (1.0)	0.03*
Cz	−6.1 (2.1)	−3.7 (0.6)	0.05*
Pz	−3.8 (2.1)	−3.0 (0.4)	0.48
dMMN latency (ms)
F3	169.3 (18.5)	182.5 (8.2)	0.19
F4	174.2 (20.1)	182.0 (8.1)	0.47
Fz	176.2 (13.6)	181.5 (8.2)	0.47
Cz	180.2 (19.7)	190.0 (13.3)	0.37
Pz	186.8 (25.2)	195.5 (23.2)	0.54
P3a amplitude (μV)
F3	1.0 (1.4)	1.5 (1.1)	0.60
F4	1.2 (2.0)	1.2 (1.1)	0.96
Fz	1.6 (1.5)	2.0 (1.2)	0.67
Cz	1.9 (1.6)	2.6 (1.4)	0.47
Pz	2.0 (1.4)	0.7 (0.8)	0.10
P3a latency (ms)
F3	264.7 (27.9)	267.0 (27.9)	0.88
F4	270.1 (31.3)	268.0 (31.3)	0.90
Fz	269.1 (32.3)	267.5 (32.3)	0.93
Cz	264.8 (28.8)	270.5 (28.8)	0.71
Pz	268.4 (29.0)	286.5 (29.0)	0.26
RON amplitude (μV)
F3	−4.3 (1.7)	−3.1 (1.2)	0.20
F4	−4.5 (1.4)	−3.1 (1.0)	0.08
Fz	−4.6 (1.6)	−2.8 (2.1)	0.08
Cz	−4.2 (2.1)	−2.5 (1.6)	0.16
Pz	−2.7 (1.8)	−2.7 (1.1)	0.97
RON latency (ms)
F3	388.0 (51.3)	353.5 (33.4)	0.22
F4	403.6 (51.4)	405.5 (72.0)	0.95
Fz	391.3 (44.8)	419.5 (53.8)	0.29
Cz	399.3 (48.6)	394.5 (28.4)	0.85
Pz	401.8 (51.3)	401.2 (33.4)	0.98

*Values represent mean (SD)*.

*^a^ Risperidone equivalent (mg/day)*.

*ARMS, at-risk mental state; Non-C., ARMS non-Converters; Conv., ARMS Converters; SAPS, Scale for the Assessment of Positive Symptoms; SANS, Scale for the Assessment of Negative Symptoms*.

*p < 0.05.

Sixteen out of 19 ARMS subjects were not taking any medication, while three were prescribed a small dose of risperidone (1.5 mg/day), aripiprazole (3 mg/day), and sulpiride (150 mg/day), respectively, for acute psychosis episodes (sometimes with strong agitation), based on the criteria of International Early Psychosis Association Writing Group (2005). MMN recordings for these subjects were conducted immediately after medications were started (9, 15, and 27 days). Two out of the three subjects subsequently developed schizophrenia. Thirteen out of 19 FES patients and 15 out of 19 CS patients were taking antipsychotic medications. There were no significant differences among ARMS, FES, and CS groups in SAPS [*F*(2,56) = 2.29, *p* = 0.11, ANOVA] and SANS [*F*(2,56) = 0.52, *p* = 0.59, ANOVA] scores. Conv and Non-C groups did not differ in the SAPS and SANS scores at baseline (*p* = 0.08, 0.24, respectively, *t*-test).

### Comparisons of ERP between healthy controls vs. ARMS vs. schizophrenia

Grand average ERP waveforms in the Fz lead following deviant stimulation are shown in Figure 1. Scatterplots of dMMN and RON amplitudes at Fz lead are shown in Figures 2A,B, respectively. P3a did not show any statistical differences so we skipped making scatterplot of P3a. ARMS subjects showed relatively smaller dMMN amplitudes at Fz (−6.5 ± 2.0 μV) compared to those of healthy control subjects (−7.4 ± 1.4 μV), which was not statistically significant (*p* = 0.13, *t*-test). On the other hand, FES group showed significantly smaller dMMN amplitudes at Fz (−5.4 ± 1.9 μV) compared to healthy control (*p* = 0.001, *t*-test). Patients with CS showed greater amplitude reductions at Fz (−4.8 ± 1.5 μV) compared to healthy controls (*p* = 0.000004, *t*-test).

At-risk mental state subjects showed relatively smaller RON amplitudes at Fz (−4.2 ± 1.8 μV) than healthy controls (−5.1 ± 1.6 μV), which was not significant (*p* = 0.15, *t*-test). On the other hand, FES group showed significantly smaller RON amplitudes at Fz (−3.9 ± 1.4 μV, *p* = 0.02). Patients with CS also elicited significantly smaller RON amplitudes at Fz (−3.4 ± 1.7 μV) compared to healthy controls (*p* = 0.005, *t*-test).

Latencies of dMMN, P3a, and RON at any electrodes did not differ among the four groups (see Table 1B).

### Comparisons between converters vs. non-converters

Grand average ERP waveforms are shown in Figure 3. Scatterplots of dMMN and RON amplitudes at Fz lead are shown in Figures 4A,B, respectively. P3a did not show any statistical differences so we skipped making scatterplot of P3a. Waveforms of Conv group were similar to those of FES patients. By contrast, waveforms of Non-C subjects resembled to those of healthy controls. Conv subjects showed significantly smaller dMMN amplitudes at Fz and Cz electrodes compared with Non-C subjects (*p* = 0.03, 0.05 by *t*-test, respectively, Table 2). On the other hand, amplitudes of Non-C did not differ from those of HC (*p* = 0.51 at Fz, *t*-test, data not shown) and there was no significant difference in dMMN amplitudes between Conv and FES subjects (*p* = 0.44 at Fz, *t*-test, data not shown). In other electrode of Non-C vs. HC and Conv vs. FES comparisons, differences were smaller and did not reach significance.

Conv subjects tended to show smaller RON amplitudes compared to those of Non-C subjects at Fz and F4 electrodes (*p* = 0.08, *p* = 0.08 by *t*-test, respectively, Table 2). Also, HC group showed relatively larger RON amplitudes at Fz lead compared to Conv subjects, which did not reach significant level (*p* = 0.08, *t*-test, data not shown). No significant differences were found at any electrode between FES vs Non-C groups (data not shown).

Latencies of dMMN, P3a, and RON at any electrodes did not differ between Conv and Non-C groups (see Table 2).

### Relationship between Symptoms and ERPs

We evaluated the correlations between dMMN, P3a, and RON amplitudes and symptoms (SAPS and SANS) in patients (schizophrenia and ARMS, *n* = 57).

Data are shown in Table 3. There were significant correlation between attention disorder score (SANS) and dMMN amplitude at Fz and F3 lead (*r* = 0.317; *p* = 0.025, *r* = 0.290, *p* = 0.041, respectively, by Pearson’s correlation). Moreover, there were significant correlation between positive formal thought disorder score (SAPS) and RON amplitude at Fz and F3 lead (*r* = 0.280; *p* = 0.049, *r* = 0.346, *p* = 0.014, respectively, by Pearson’s correlation). Thus, reduction of ERPs was correlated with severity of some symptoms.

**Table 3:** **ERP amplitudes and symptoms**.

	SAPS							
	Hallucinations		Delusions		Bizarre behavior		Positive formal thought disorder	
	*r*	*p*	*r*	*p*	*r*	*p*	*r*	*p*
dMMN amplitude (μV)										
F3	0.039	0.786	0.011	0.938	−0.122	0.398	0.086	0.552
F4	0.071	0.625	0.066	0.648	−0.131	0.365	0.076	0.599
Fz	0.021	0.884	−0.016	0.910	−0.199	0.166	0.090	0.536
Cz	−0.021	0.888	−0.036	0.805	−0.108	0.457	0.056	0.697
Pz	−0.163	0.258	−0.178	0.216	−0.225	0.117	−0.069	0.636
P3a amplitude (μV)										
F3	−0.148	0.305	−0.188	0.192	−0.188	0.191	−0.036	0.802
F4	−0.075	0.605	−0.190	0.187	−0.256	0.073	0.029	0.842
Fz	−0.191	0.185	−0.181	0.209	−0.233	0.104	−0.008	0.956
Cz	−0.149	0.302	−0.056	0.701	−0.213	0.138	0.020	0.891
Pz	0.022	0.879	0.046	0.753	−0.023	0.874	−0.117	0.417
RON amplitude (μV)										
F3	0.014	0.926	−0.131	0.363	0.067	0.646	0.280	0.049*
F4	0.087	0.549	−0.092	0.523	−0.158	0.274	0.244	0.087
Fz	−0.024	0.869	−0.109	0.450	−0.265	0.063	0.346	0.014*
Cz	−0.033	0.818	−0.214	0.136	−0.081	0.578	0.151	0.295
Pz	0.002	0.990	−0.257	0.071	−0.025	0.861	0.022	0.881

	SANS									
	Affective flattening		Alogia		Avolition- apathy		Anhedonia- asociality		Attention	
	*r*	*p*	*r*	*p*	*r*	*p*	*r*	*p*	*r*	*p*

dMMN amplitude (μV)										
F3	0.109	0.452	0.149	0.301	0.096	0.509	−0.102	0.483	0.317	0.025*
F4	0.142	0.325	0.122	0.399	0.016	0.910	−0.066	0.650	0.260	0.068
Fz	0.115	0.427	0.165	0.254	−0.014	0.923	−0.081	0.576	0.290	0.041*
Cz	0.060	0.680	0.147	0.307	0.117	0.420	0.050	0.730	0.262	0.066
Pz	−0.041	0.778	0.066	0.650	0.122	0.400	−0.063	0.666	−0.007	0.963
P3a amplitude (μV)										
F3	−0.029	0.843	−0.034	0.815	0.037	0.796	−0.037	0.796	0.130	0.368
F4	0.021	0.883	0.021	0.885	0.003	0.984	−0.102	0.480	0.148	0.306
Fz	−0.043	0.767	0.032	0.823	−0.032	0.827	−0.090	0.533	0.101	0.487
Cz	−0.066	0.649	−0.029	0.843	0.012	0.934	−0.010	0.943	0.112	0.441
Pz	0.063	0.662	−0.027	0.852	0.108	0.454	−0.046	0.753	0.032	0.827
RON amplitude (μV)										
F3	−0.112	0.438	−0.111	0.441	−0.054	0.712	−0.215	0.134	−0.055	0.704
F4	0.022	0.882	0.039	0.788	−0.089	0.539	−0.103	0.475	−0.050	0.730
Fz	−0.046	0.752	−0.017	0.905	−0.104	0.474	−0.073	0.617	−0.025	0.861
Cz	0.128	0.375	0.210	0.143	−0.040	0.781	0.002	0.988	−0.108	0.455
Pz	0.014	0.922	0.095	0.513	−0.123	0.393	−0.117	0.419	−0.242	0.090

*SAPS, Scale for the Assessment of Positive Symptoms; SANS, Scale for the Assessment of Negative Symptoms*.

*p < 0.05*, r* = Pearson product–moment correlation coefficient.

## Discussion

Duration mismatch negativity amplitudes at frontal and central leads were reduced in ARMS subjects who later converted to overt schizophrenia in comparison with non-converters and normal subjects, consistent with previous reports (Bodatsch et al., 2011; Shaikh et al., 2012; Higuchi et al., 2013b). Specifically, the current data from gender matched subjects across groups (Table 1) confirmed previous observations in patients with variable demographic backgrounds (Bodatsch et al., 2011; Shaikh et al., 2012; Higuchi et al., 2013b). Importantly, this study is the first to suggest that RON provides a marker for the progression to overt schizophrenia in subjects with ARMS, based on longitudinal observations.

Three out of 4 Conv, 7 out of 15 Non-C, 7 out of 19 FES, 5 out of 19 CS, and 9 out of 19 HC subjects overlapped with subjects in our previous report (Higuchi et al., 2013b). We selected subjects for the current study, according to the following considerations; (1) ARMS subjects with a longer followed-up period, (2) gender-match between HC and schizophrenia patients, (3) younger HC and schizophrenia patients than those used in the previous study. The current one used a longer observation period, and was gender-matched across groups with less variation in age. According to a previous report (Yung et al., 2003), 10–40% of ARMS subjects later developed schizophrenia, consistent with our observations that 21.0% progressed to the illness.

ARMS subjects as a whole have been reported to demonstrate reduced dMMN amplitudes, but with a lesser degree compared to patients with overt schizophrenia (Bodatsch et al., 2011; Atkinson et al., 2012; Jahshan et al., 2012), consistent with the present results (Figure 1). On the other hand, the current data may be partly different from our previous observations indicating the lack of difference in dMMN amplitudes between ARMS subjects as whole and healthy controls (Higuchi et al., 2013b). One of the reasons for this discrepancy may include the difference in age and gender ratio. In fact, as previous reports indicate ERPs amplitudes gradually decrease by age, and male subjects show relatively smaller amplitudes than female because of the difference in skull thickness (Ikezawa et al., 2008; Matsubayashi et al., 2008; Naatanen et al., 2012). Another confounding factor may include the observation periods for follow-up. While our previous report (Higuchi et al., 2013b) employed a relatively short period (mean ± SD = 1.6 ± 0.8 years for non-converters), the present study used a longer period (2.2 ± 1.5 years), similar to those in the literature.

Compared to Non-C, Conv subjects elicited significantly smaller dMMN amplitudes at F4 and Fz leads (Table 2). These observations suggest the ability of dMMN amplitudes to differentiate between high-risk individuals who later progress to schizophrenia and those who do not, as has been suggested (Higuchi et al., 2013b; Sumiyoshi et al., 2013).

Little information has been available about the feature of RON in schizophrenia. In this study, RON amplitudes of ARMS subjects as a whole were not different from those of HC subjects, while FES and CS group showed significantly smaller RON amplitudes at Fz and F4 leads compared to the HC group. This finding is consistent with observations by Jahshan et al. (2012). As the results of the current study suggest that RON amplitudes may decrease according to progression of clinical stages of schizophrenia (Table 1B; Figure 1), they may provide an intermediate phenotype of the illness.

Importantly, RON amplitudes of Conv subjects tended to be smaller than those of Non-C at the Fz and F4 leads (Figure 4). The failure to reach statistical significance may be due to the fact that RON waveforms are not stable and smaller compared to dMMN waveforms. Future investigations with a larger number of subjects would be desirable to determine if the combined measurement of RON and dMMN would further facilitate early detection of schizophrenia.

P3a amplitudes were barely detectable in this study (Figures 1 and 3). These amplitudes have been reported to be decreased in schizophrenia and ARMS (Friedman et al., 2001; Jahshan et al., 2012; Mondragon-Maya et al., 2013; Nagai et al., 2013). Variations of P3a amplitudes may be large, due, probably, to the difference in measurement.

Limitations of this study include the small sample number, especially in ARMS (*n* = 19) and Conv subjects (*n* = 4). According to the power analysis, at least 26 patients are needed to obtain adequate effect size (i.e., 0.6). Investigations with a larger number of patients will make the data more satisfactory. Second, significant age difference was seen in the ARMS vs. HC and FES vs. CS comparisons. Since part of ARMS subjects is regarded as prodromal state of schizophrenia, it is natural that they are mostly younger than schizophrenia patients. Therefore, adjustment of age between FES/CS and ARMS subjects may increase the number of certain type of schizophrenia, e.g., hebephrenic type. Due to an effort to make the FES/CS groups more homogeneous, patients of these groups became somewhat older than the ARMS group. Application of ANCOVA to 19 members may provide over-adjustment. Although MMN amplitudes are reduced gradually by age, the decline is not substantial (−0.056 μV/year in schizophrenia and −0.079 μV/year in healthy control) (Kiang et al., 2009). ARMS/HC subjects are about 2.5 years younger than FES/CS (Table 1). According to this formula, about 0.2 μV amplitude reduction may occur between these two. Differences in our data presented (at Fz lead) were 1.1 μV or greater (ARMS vs. FES groups.), which was sufficiently large. Third, some ARMS subjects and most schizophrenia patients were taking antipsychotic drugs, which may be another limitation of the current study. Fourth, in this study, we measured ERPs at baseline, and did not perform follow-up measurements. Therefore, little information is available about longitudinal data of ERPs parameters.

In conclusions, diminished amplitudes in dMMN/RON may provide a biomarker that is present before and after the development of psychosis. Our results should be interpreted with caution before applying to the at-risk population, especially to avoid over-diagnosis. Ideally, the combination with other cognitive modalities, e.g., neuropsychological tests (Higuchi et al., 2013b), brain morphology, and biochemical markers, would enhance the sensitivity and specificity for early diagnosis. These efforts are expected to help improve functional outcome in subjects with schizophrenia and vulnerable individuals as well.

## Conflict of Interest Statement

The authors declare that the research was conducted in the absence of any commercial or financial relationships that could be construed as a potential conflict of interest.
